# Prognostic Value of Cardiac Troponin and Risk Assessment in Pediatric Supraventricular Tachycardia

**DOI:** 10.3390/jcm10163638

**Published:** 2021-08-17

**Authors:** Chieh-Ching Yen, Shou-Yen Chen, Chung-Hsien Chaou, Chih-Kai Wang, Hsin-Tzu Yeh, Chip-Jin Ng

**Affiliations:** 1 Department of Emergency Medicine, Chang Gung Memorial Hospital, Linkou Branch, Taoyuan City 333, Taiwan; chiehching74@gmail.com (C.-C.Y.); shien@url.com.tw (C.-H.C.); zero88015@hotmail.com (C.-K.W.); yeh7504@gmail.com (H.-T.Y.); ngowl@ms3.hinet.net (C.-J.N.); 2 College of Medicine, National Yang Ming University, Taipei City 112, Taiwan; 3 Chang Gung Medical Education Research Center, Chang Gung Memorial Hospital, Taoyuan City 333, Taiwan; 4 Division of Medical Education, Graduate Institute of Clinical Medical Sciences, College of Medicine, Chang Gung University, Taoyuan City 333, Taiwan; 5 College of Medicine, Chang Gung University, Taoyuan City 333, Taiwan

**Keywords:** supraventricular tachycardia, cardiac troponin, pediatric patients, emergency department

## Abstract

Cardiac troponin I (cTnI) elevation is common in an acute episode of supraventricular tachycardia (SVT). However, there is limited evidence regarding the prognostic value of cTnI and the predictors of SVT recurrence in pediatric patients. We screened the electronic medical records of all pediatric patients presenting to the emergency departments at five Taiwanese hospitals from 1 January 2010 to 31 May 2021. Our primary outcomes were the occurrence of major adverse cardiac events (MACEs) during the follow-up period and 30-day SVT recurrence. A total of 112 patients were included in our study. Of these, 29 (25.9%) patients had positive cTnI values. Patients with cTnI elevation had significantly more complaints of dyspnea (27.6% vs. 7.2%, *p* = 0.008) and gastrointestinal discomfort (24.1% vs. 4.8%, *p* = 0.006). There were significantly more intensive care unit admissions (41.4% vs. 16.9%, *p* = 0.007) among the cTnI-positive group. One MACE was found in the cTnI-negative group. For 30-day SVT recurrence, the cTnI-positive group had a higher recurrence rate, without a statistically significant difference (20.7% vs. 7.2%, *p* = 0.075). Multivariable logistic regression analysis showed hypotension as an independent predictor of 30-day SVT recurrence (OR = 4.98; Cl 1.02–24.22; *p* = 0.047). Troponin had low value for predicting the outcomes of pediatric patients with SVT. The only significant predictor for recurrent SVT was initial hypotension.

## 1. Introduction

Supraventricular tachycardia (SVT) is the most common group of arrhythmias in the pediatric population, with an incidence of up to 1 in 250 individuals [1]. There are various symptoms in pediatric patients, ranging from gastrointestinal, respiratory, to psychosomatic presentations, which are somewhat different from those of adult patients [2,3,4,5]. The diagnosis of SVT can be made by electrocardiogram and electrophysiologic studies by mapping the electrical conduction systems and identifying aberrant pathways [2].

Cardiac enzymes, such as troponin, play important roles in the diagnosis and treatment of acute coronary syndrome (ACS) [6]. A number of studies have shown that elevated troponin levels are observed in other acute heart and vessel diseases, such as aortic dissection, pericarditis, myocarditis, and pulmonary embolism [6,7,8,9]. The value of troponin in predicting prognosis in SVT patients remains inconclusive. Although some research has demonstrated an increased risk for future adverse outcomes in specific populations, there have been few studies on the utility of troponin and recurrence risk in pediatric patients with SVT [10,11,12]. The aims of our study were to evaluate the utility of troponin in pediatric patients with SVT and to identify the predictors of SVT recurrence.

## 2. Materials and Methods

### 2.1. Study Design and Setting

This was a retrospective, multiple-center, observational study. The study was conducted at five Taiwanese hospitals, including two tertiary care centers and three regional hospitals, with a total capacity of 9000 beds and 500,000 annual emergency department (ED) visits. The study period was between 1 January 2010 and 31 May 2021. This study was approved by the Chang Gung Medical Foundation Institutional Review Board (IRB no. 202100905B0) and qualified for a waiver of informed consent.

### 2.2. Patient Selection and Data Collection

**Figure 1:**
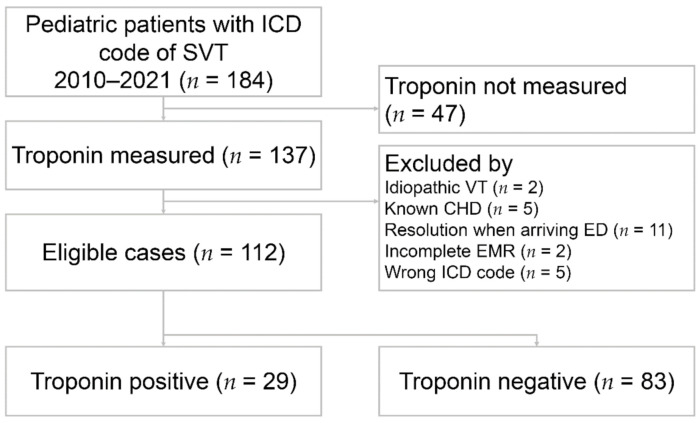
Flow chart of patient selection. ICD: International Classification of Diseases; SVT: supraventricular tachycardia; VT: ventricular tachycardia; CHD: congenital heart disease; ED: emergency department; EMR: electrical medical record.

We first identified all pediatric patients aged 0–18 years with International Classification of Diseases (ICD)-9 code 427.0 and ICD-10 code I471 of supraventricular tachycardia (SVT) who presented to the ED through a computerized search from the electrical medical record (EMR) system. The patients selected by EMR were reviewed for 12-lead electrocardiogram and documentation by attending physicians. Patients with a confirmed diagnosis of SVT and troponin tests were included. Patients with known congenital heart disease, incomplete medical records, arrhythmias other than SVT, or resolution of SVT when arriving at the ED were excluded through a detailed chart review (Figure 1). SVT was defined as regular narrow complex tachycardia but did not include atrial fibrillation and flutter. The diagnosis of SVT was confirmed by 12-lead electrocardiogram and documentation in the ED notes of attending physicians.

We collected the relevant variables, including age, sex, vital signs, symptoms and signs, laboratory results, electrocardiograms, chest radiographs, length of hospital stay, underlying diseases, medication therapies, whether patients received radiofrequency ablation, and ED disposition. A peak troponin value was defined as the highest value if patients underwent follow-up troponin testing. Further cardiac investigations, including echocardiography and electrophysiological (EP) studies, were also documented.

The primary outcomes were the occurrence of major adverse cardiac events (MACEs) during the follow-up period and 30-day SVT recurrence. MACE was defined as ischemic stroke, admission due to acute decompensated heart failure, acute coronary syndrome, revascularization, coronary artery bypass grafting (CABG), and all-cause mortality.

### 2.3. Measurement of Cardiac Troponin I

The cardiac troponin I (cTnI) levels were measured with a UniCel DxI 800 immunoassay analyzer (Beckman Coulter DxC880i, Danaher Corporation, Brea, California, United States). The assay has a minimum detectable concentration of <0.01 ng/mL, and the cutoff level (coefficient of variation of ≤10%) for positivity is 0.04 ng/mL.

### 2.4. Statistical Analysis

Descriptive data are presented as the means ± *SD*s or as numbers (percentages). Categorical data were analyzed with chi-square or Fisher’s exact tests, as appropriate, and continuous variables were analyzed using independent Student’s *t*-tests for normally distributed continuous variables and Mann–Whitney U-tests for skewed continuous variables. Multivariable logistic regression was performed for outcome risk assessment with stepwise backward selection of variables, with a *p*-value cutoff of 0.1. As a result of normal variations of baseline blood pressure and heart rate in pediatric patients, hypotension was defined as systolic blood pressure lower than 2× age (years) + 70 mmHg, and heart rate was transformed to an age-adjusted *z* score [13]. All *p*-values were two-sided, and the significance level was set at 0.05. All statistical analyses were performed using SPSS software (version 13.0 for Windows; SPSS Inc., Chicago, IL, USA).

## 3. Results

### 3.1. Patient Characteristics

During the study periods, a total of 112 patients were included in our study. Of these, 29 (25.9%) patients had a positive cardiac troponin I (cTnI) value, and 83 (74.1%) had a negative value. Age and male proportion were similar between the two groups. The unadjusted peak heart rate was significantly higher in the cTnI-positive group (219 vs. 200.8 beats per min, *p* = 0.037), but no significant difference was found after adjusting the heart rate with the *z* score (6.5 vs. 6.3, *p* = 0.537). For symptoms, patients with cTnI elevation had significantly more complaints of dyspnea (27.6% vs. 7.2%, *p* = 0.008) and gastrointestinal discomfort (24.1% vs. 4.8%, *p* = 0.006). There was no difference in the medical treatment of SVT between the two groups. Among the cTnI-positive group, there were significantly more intensive care unit admissions (41.4% vs. 16.9%, *p* = 0.007). The length of hospital stay was longer in the cTnI-positive group, without a significant difference (48.3 vs. 28.4 h, *p* = 0.19). The detailed patient characteristics are summarized in Table 1.

For laboratory investigations, the results were mostly within normal ranges. None of our patients with hemoglobin testing received blood transfusions. Electrolyte levels, including sodium, potassium, calcium, and magnesium, were almost normal. Only one patient in the cTnI-negative group had a free thyroxine level of 4.75 ng/dL (normal range: 0.7–1.48 ng/dL) and was diagnosed with primary hyperthyroidism. The full laboratory results are reported in Table 2.

Mechanisms of SVT were divided into atrioventricular reentrant tachycardia (AVRT), atrioventricular nodal reentrant tachycardia (AVNRT), and atrial tachycardia (AT) according to EP studies, while Wolff–Parkinson–White syndrome (WPW syndrome) was classified on the basis of electrocardiogram findings with the characteristic delta wave of ventricular pre-excitation. No significant difference was noted between the two groups (Table 3). Echocardiography was performed in most patients (*N* = 95, 81.2%). Among patients with parameter data (*N* = 56), left atrial diameter was non-significantly larger in the cTnI-negative group (23.20 vs. 20.56 mm, *p* = 0.093). Two (7.4%) patients in the cTnI-positive group had abnormal findings: one had a small muscle ridge over the middle portion of the interventricular septum without left ventricular outflow tract obstruction, and the other had a stationary right atrium cardiac mass. Five (7.4%) patients in the cTnI-negative group had abnormal findings: one had pre-existing Pompe disease-related hypertrophic cardiomyopathy, one had a ventricular septal defect, one had congestive heart failure with moderate mitral regurgitation and tricuspid regurgitation, and two had mitral valve prolapse.

### 3.2. Clinical Outcomes

During the follow-up period (40 ± 37 months), one major adverse cardiac event (MACE) was found in the cTnI-negative group. The patient was hospitalized due to acute decompensated heart failure six months after the first presentation of SVT at our ED. All patients survived until the last follow-up time. For 30-day SVT recurrence, the cTnI-positive group had a higher recurrence rate, without a statistically significant difference (20.7% vs. 7.2%, *p* = 0.075) (Table 4).

**Figure 2:**
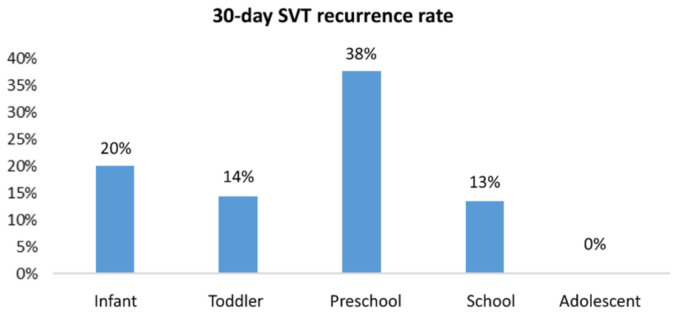
Distribution of 30-day SVT recurrence rate by age group. Infant: 28 d—<1 y; Toddler: 1—<3 y; Preschool: 3—<6 y; School: 6—<13 y; Adolescent: 13—≤18 y.

**Figure 3:**
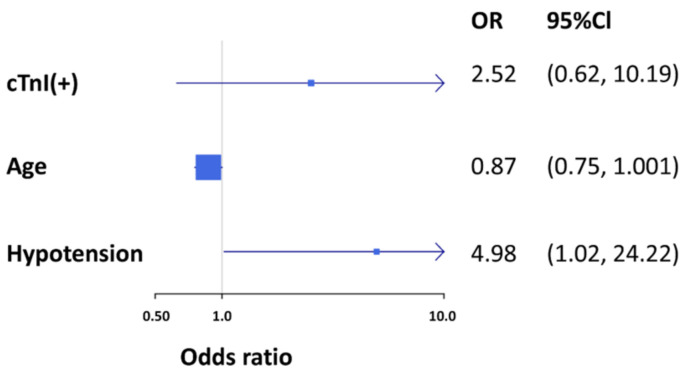
Multivariable predictors of 30-day SVT recurrence.

The age distribution of 30-day SVT recurrence is shown in Figure 2. No 30-day SVT recurrence was found in patients aged 13 years or older, which had a sensitivity of 100% and specificity of 60%. The results of univariate and multivariable logistic regression analyses showed hypotension as an independent predictor of 30-day SVT recurrence (OR = 4.98; Cl 1.02–24.22; *p* = 0.047) (Table 5) (Figure 3).

## 4. Discussion

We have presented, to the best of our knowledge, the largest cohort study evaluating the prognostic value of troponin and comprehensive laboratory investigations in pediatric patients with supraventricular tachycardia (SVT). This study also offers the first description of hypotension as a predictor for 30-day SVT recurrence.

A prior adult systemic review and meta-analysis investigated the prevalence of troponin elevation in patients presenting with SVT and revealed a pooled proportion of 32% (12–46%) [11]. One pediatric study illustrated a prevalence of 29% in patients with SVT at the time of the index ED presentation, which showed a similar proportion of elevated troponin levels [5]. It is not uncommon that troponin elevation occurs in the absence of coronary artery disease after SVT presentation [10,11,14,15,16]. However, the utility of troponin in patients with SVT is still debated [17].

As recommended by consensus guidelines, troponin is used as a biomarker for acute myocardial infarction (MI) diagnosis, accompanied by symptoms and electrocardiographic (ECG) findings [18]. However, limited evidence reveals the diagnostic value of troponin in SVT. One study conducted by Moore J.P. et al. analyzed the predictors of troponin elevation in pediatric SVT patients, and 13 patients with elevated troponin levels were all alive at the time of the last follow-up [5]. Nevertheless, there were no outcome variables, for instance, with rehospitalization, cardiac intervention, and SVT recurrence shown in their study. Our study indicated that pediatric SVT patients with positive troponin values had similar outcomes compared with patients with normal troponin levels. Troponin also had a poor predictive value for 30-day SVT recurrence. Troponin provides little prognostic significance and may contribute to unnecessary admissions, longer length of stay, and higher health care costs.

It remains unclear which mechanisms contribute to the elevation of troponin in SVT patients. In the adult literature, possible precipitating factors include concurrent microvascular coronary disease, occult cardiomyopathy, or demand ischemia-related myocyte injury [19,20,21]. Given the low prevalence rates of coronary artery disease and cardiomyopathy in pediatric patients, the pathophysiology of elevated troponin levels in SVT might differ from that in adult patients. The increase in heart rate during SVT results in shortening of diastole with decreased oxygen supply, and simultaneous increased oxygen consumption may lead to subendocardial ischemia. Another feature of troponin elevation during tachycardia is myocardial stretch, which was identified to be associated with myocyte functional injury and cell death [22]. A close relationship has been demonstrated between an increase in troponin and B-type natriuretic peptide (BNP) in patients with various tachyarrhythmias [23]. These reports implied that increased levels of troponin could probably be attributed to the physiology of SVT and were not associated with major cardiovascular events in pediatric patients.

It has been demonstrated that congenital heart disease (CHD) is related to infants with SVT. Studies have shown prevalence rates of 30% of CHD in SVT infants with WPW syndrome and 20% in those without WPW syndrome [1,24,25]. However, one study conducted by L’Italien, K., et al. revealed that the prevalence of CHD in patients aged two or older presenting with SVT was similar to that in the general population, and only one patient in the cohort required immediate intervention [1]. Our study showed similar results, and no patients needed to receive emergent operations. Although left atrial diameter was non-significantly larger in the cTnI-negative group, it may be less meaningful given the combined influence of weight, height, and age on cardiac size and the limited parameter data of our patients [26]. Immediate echocardiography seems unnecessary in the acute setting.

Blood tests, including hemoglobin, electrolytes, and thyroid function tests, were common in patients who underwent SVT. Anemia was recognized as a possible risk factor for SVT, and it should be considered if other symptoms of anemia exist [27]. Abnormal potassium and magnesium levels were reported to lead to arrhythmias, but no direct evidence supports the relationship between electrolyte imbalance and SVT [11,27,28]. Thyroid function tests were recommended in SVT patients by the UK National Service Framework guidelines [11,27]. Although there is no well-established evidence regarding the association between thyroid dysfunction and SVT, a few case reports have demonstrated that either hyperthyroidism or hypothyroidism may lead to SVT by inducing sympathetic-vagal imbalance [29,30,31].

There have been few documented risk factors for SVT recurrence in pediatric patients in previous studies. Hypotension on initial presentation was found to be an independent predictor for recurrent SVT 30 days after adjustment for associated variables in our study. Another interesting finding was that none of the patients aged 13 or older had SVT recurrence within 30 days. Although one previous study reported that patients with an onset age younger than one year were at decreased risk of recurrence at the final follow-up, no other research mentioned age as an indicator for predicting SVT recurrence [32]. These findings may help physicians determine the best course of action in the ED. For patients at risk of SVT recurrence, close outpatient department follow-up and further cardiac investigations, such as EP studies, are appropriate.

This study has several limitations, which need to be mentioned. It was a retrospective study, and selection bias may exist. Some clinical variables, such as detailed symptoms and definite onset time of SVT, could be missed if not recorded in the EMR. Second, given that not every patient underwent further EP studies, the exact mechanism of SVT in one-half of the patients was unknown. Finally, the small number of patients in our study may limit the overall power and hamper our ability to draw clear-cut conclusions, although, to our knowledge, this was the largest study on the topic.

## 5. Conclusions

Troponin had low value for predicting outcomes of pediatric patients with SVT. The only significant predictor for recurrent SVT was initial hypotension. Emergent blood tests and echocardiography may not be necessary in the ED and could be performed at follow-up.

## Figures and Tables

**Table 1:** Patient characteristics according to cTnI levels.

Variable	cTnI(+) (*n* = 29)	cTnI(–) (*n* = 83)	*p* Value
Age (yr)	8.6 ± 5.4	10.3 ± 4.7	0.099
Male	13 (44.8%)	37 (44.6%)	1.000
Previous SVT	10 (34.5%)	30 (36.1%)	1.000
Vital signs			
Peak heart rate (beats/min)	219.0 ± 42.0	200.8 ± 28.5	0.037
Peak heart rate (*z* score)	6.5 ± 1.6	6.3 ± 1.5	0.537
Systolic blood pressure (mmHg)	101.5 ± 18.9	108.9 ± 18.5	0.084
Diastolic blood pressure (mmHg)	64.1 ± 12.0	70.8 ± 14.0	0.027
Hypotension	6 (21.4%)	7 (9.2%)	0.106
Fever ^†^	2 (6.9%)	11 (13.3%)	0.509
Symptoms			
Palpitation	23 (79.3%)	69 (83.1%)	0.779
Chest pain	7 (24.1%)	18 (21.7%)	0.799
Dyspnea	8 (27.6%)	6 (7.2%)	0.008
Dizziness	2 (6.9%)	8 (9.6%)	1.000
Syncope	0 (0%)	2 (2.4%)	1.000
Respiratory	7 (24.1%)	12 (14.5%)	0.257
Gastrointestinal	7 (24.1%)	4 (4.8%)	0.006
SVT duration since ED (min)	60.5 ± 80.0	46.8 ± 78.7	0.424
Length of stay (hr)	48.3 ± 111.5	28.4 ± 47.0	0.190
Treatment			
Without medication ^§^	3 (10.3%)	11 (13.4%)	1.000
Adenosine use	25 (86.2%)	72 (86.7%)	1.000
Verapamil use	1 (3.4%)	4 (4.8%)	1.000
Amiodarone use	5 (17.2%)	10 (12.0%)	0.530
Radiofrequency ablation ^‡^	16 (34.0%)	1 (10.0%)	0.247
ED Disposition			
Ordinary medical ward	6 (20.7%)	21 (25.3%)	0.802
Intensive care unit	12 (41.4%)	14 (16.9%)	0.007
Discharge	11 (37.9%)	48 (57.8%)	0.084

Count data are expressed as number (percentage) and continuous values are expressed as mean ± *SD*. cTnI: cardiac troponin I; SVT: supraventricular tachycardia; ED: emergency department. ^†^ Defined as body temperature > 38 °C in the triage, ^§^ Defined as spontaneous resolution or Valsalva maneuver, ^‡^ Performed within five years after patients discharge.

**Table 2:** Analysis of laboratory findings in patients with or without elevated cTnI levels.

Variable	cTnI(+) (*n* = 29)	cTnI(–) (*n* = 83)	*p* Value
Laboratory exam			
White cell count (10^3^/uL) (*n* = 107)	12.45 ± 5.41 ^‡^	10.05 ± 3.57	0.01
Hemoglobin (g/dL) (*n* = 107)	13.48 ± 1.41	13.56 ± 1.34	0.775
Platelet (10^3^/uL) (*n* = 107)	316.1 ± 89.1	285.9 ± 68.0	0.06
Blood urea nitrogen (mg/dL) (*n* = 41)	12.8 ± 2.8	13.2 ± 10.5	0.908
Creatinine (mg/dL) (*n* = 74)	0.56 ± 0.20	0.69 ± 1.17	0.622
Sodium (mEq/L) (*n* = 94)	139.6 ± 2.2	139.4 ± 2.2	0.733
Potassium (mEq/L) (*n* = 93)	4.12 ± 0.56	3.95 ± 0.40	0.109
Calcium (mg/dL) (*n* = 70)	9.47 ± 0.40	9.38 ± 0.52	0.488
Magnesium (mEq/L) (*n* = 39)	1.85 ± 0.18	1.80 ± 0.20	0.499
AST (U/L) (*n* = 70)	32.50 ± 16.07	38.18 ± 46.41	0.596
ALT (U/L) (*n* = 62)	21.69 ± 16.49	31.26 ± 48.14	0.441
Initial cTnI (ng/mL) (*n* = 112)	0.172 ± 0.163 ^‡^	0.015 ± 0.010	<0.001
Peak cTnI (*n* = 112)	0.256 ± 0.299 ^‡^	0.017 ± 0.008	<0.001
TSH (uIU/mL) (*n* = 23)	2.37 ± 1.41	2.31 ± 1.52	0.926
Free thyroxine (ng/dL) (*n* = 20)	1.26 ± 0.17	1.57 ± 1.15	0.436
BNP (pg/mL) (*n* = 19)	173.4 ± 185.3 ^‡^	163.3 ± 232.9 ^‡^	0.927
C-Reactive Protein (mg/L) (*n* = 24)	5.07 ± 9.23 ^‡^	1.13 ± 1.64	0.105

Continuous values are expressed as mean ± *SD*. cTnI: cardiac troponin I; AST: aspartate aminotransferase; ALT: alanine aminotransferase; TSH: thyroid-stimulating hormone; BNP: B-type natriuretic peptide; ^‡^ Increased value.

**Table 3:** Comparisons of SVT mechanisms and echocardiographic findings in patients with or without elevated cTnI levels.

Variable	cTnI(+) (*n* = 29)	cTnI(–) (*n* = 83)	*p* Value
SVT mechanism			
AVRT ^§^	4 (13.8%)	3 (3.6%)	0.073
AVNRT	2 (6.9%)	14 (16.9%)	0.232
AT	0 (0%)	2 (2.4%)	1.000
WPW	8 (27.6%)	12 (14.5%)	0.157
Not classified by EP study	15 (51.7%)	52 (62.7%)	0.38
Echocardiography ^†^	(*n* = 27)	(*n* = 68)	
Normal	25 (92.6%)	59 (86.8%)	0.723
Incidental ^‡^	0 (0%)	4 (5.9%)	0.575
Abnormal	2 (7.4%)	5 (7.4%)	1.000
LVEF(%)	71.83 ± 5.18	70.66 ± 8.91	0.605
LA diameter (mm)	20.56 ± 6.26	23.20 ± 4.78	0.093
LVEDD (mm)	34.28 ± 10.77	38.22 ± 8.95	0.160
LVESD (mm)	20.67 ± 5.96	22.36 ± 5.55	0.307
Aortic root diameter (mm)	20.17 ± 5.85	21.11 ± 4.31	0.506

Count data are expressed as number (percentage) and continuous values are expressed as mean ± *SD*. AVRT: atrioventricular reentrant tachycardia; AVNRT: atrioventricular nodal reentrant tachycardia; AT: atrial tachycardia; WPW: Wolff–Parkinson–White syndrome; EP: electrophysiologic; LVEF: left ventricular ejection fraction; LVEDD: left ventricular end-diastolic diameter; LVESD: left ventricular end-systolic diameter. ^†^ Parameter data available in 56 patients (*n* = 18 in cTn+; *n* = 38 in cTn−). ^§^ Not including WPW syndrome. ^‡^ Incidental findings, not classified as abnormal echocardiography, included patent foramen ovale and left-sided aortic arch.

**Table 4:** Clinical outcomes of patients with positive or negative cTnI.

Variable	cTnI(+) (*n* = 29)	cTnI(–) (*n* = 83)	*p* Value
MACE	0 (0%)	1 (0.01%)	1.000
30-day SVT recurrence	6 (20.7%)	6 (7.2%)	0.075

Count data are expressed as number (percentage). MACE: major adverse cardiovascular event.

**Table 5:** Univariate and multivariable logistic regression results for 30-day SVT recurrence.

	Univariate		Multivariable	
	OR (95%CI)	*p* Value	OR (95%CI)	*p* Value
Age	0.86 (0.75, 0.98)	0.022	0.87 (0.75, 1.001)	0.051
Male	1.27 (0.38, 4.22)	0.693		
Peak heart rate (*z* score)	0.99 (0.66, 1.49)	0.965		
Hypotension	5.33 (1.31, 21.8)	0.020	4.98 (1.02, 24.22)	0.047 *
cTnI(+)	3.35 (0.99, 11.4)	0.053	2.52 (0.62, 10.19)	0.195

* *p* value < 0.05.

## Data Availability

The data in this study are available from the corresponding author upon reasonable request.
